# Theta brain rhythms index perceptual narrowing in infant speech perception

**DOI:** 10.3389/fpsyg.2013.00690

**Published:** 2013-10-11

**Authors:** Alexis N. Bosseler, Samu Taulu, Elina Pihko, Jyrki P. Mäkelä, Toshiaki Imada, Antti Ahonen, Patricia K. Kuhl

**Affiliations:** ^1^Institute for Learning & Brain Sciences, University of Washington, SeattleWA, USA; ^2^Cognitive Brain Research Unit, University of HelsinkiHelsinki, Finland; ^3^Elekta OyHelsinki, Finland; ^4^Brain Research Unit, O.V. Lounasmaa Laboratory, School of Science, Aalto UniversityHelsinki, Finland; ^5^BioMag Laboratory, HUS Medical Imaging Center, Helsinki University Central HospitalHelsinki, Finland

**Keywords:** speech perception, infants, magnetoencephalography, perceptual narrowing, brain rhythms

## Abstract

The development of speech perception shows a dramatic transition between infancy and adulthood. Between 6 and 12 months, infants' initial ability to discriminate all phonetic units across the world's languages narrows—native discrimination increases while non-native discrimination shows a steep decline. We used magnetoencephalography (MEG) to examine whether brain oscillations in the theta band (4–8 Hz), reflecting increases in attention and cognitive effort, would provide a neural measure of the perceptual narrowing phenomenon in speech. Using an oddball paradigm, we varied speech stimuli in two dimensions, stimulus frequency (frequent vs. infrequent) and language (native vs. non-native speech syllables) and tested 6-month-old infants, 12-month-old infants, and adults. We hypothesized that 6-month-old infants would show increased relative theta power (RTP) for frequent syllables, regardless of their status as native or non-native syllables, reflecting young infants' attention and cognitive effort in response to highly frequent stimuli (“statistical learning”). In adults, we hypothesized increased RTP for non-native stimuli, regardless of their presentation frequency, reflecting increased cognitive effort for non-native phonetic categories. The 12-month-old infants were expected to show a pattern in transition, but one more similar to adults than to 6-month-old infants. The MEG brain rhythm results supported these hypotheses. We suggest that perceptual narrowing in speech perception is governed by an implicit learning process. This learning process involves an implicit shift in attention from frequent events (infants) to learned categories (adults). Theta brain oscillatory activity may provide an index of perceptual narrowing beyond speech, and would offer a test of whether the early speech learning process is governed by domain-general or domain-specific processes.

## Introduction

Exposure to language early in infancy produces a dramatic shift in speech perception between 6 and 12 months, a period that has been referred to as a “critical” or “sensitive” period for the development of native-language phonetic perception (Kuhl, 2011; Peña et al., 2012). Infants begin life perceiving phonetic differences used to distinguish words across all languages, but by 12 months of age infants show a narrowing in their speech perception abilities—performance on native sound discrimination increases while at the same time performance on non-native sound discrimination declines (Werker and Lalonde, 1988; Kuhl et al., 2006).

Perceptual narrowing during the second half of the first year of life is neither restricted to speech nor to auditory stimuli: It has been demonstrated for sign language (Palmer et al., 2012), for visual stimuli such as faces (Lewkowicz and Ghazanfar, 2006), and for non-speech auditory patterns in music (Saffran et al., 1999; Saffran and Griepentrog, 2001), suggesting that perceptual narrowing may involve a pan-sensory developmental process, with sensory and cognitive effects (Lewkowicz and Ghazanfar, 2006).

Developments in cognitive neuroscience have identified brain measures that can be used to provide different kinds of information about how listeners process auditory stimuli. For example, the classic mismatch response obtained with the oddball paradigm, measured using EEG or MEG signal changes (e.g., Näätänen et al., 1997), indexes a listener's ability to discriminate two auditory stimuli. In contrast, brain oscillatory activity, measured using either electrophysiological (EEG) or magnetic (MEG) data, is being used to track brain rhythms in distinct frequency bands—alpha, theta, gamma, and beta. Power and phase variations in these frequency bands are associated with psychological processes including attention, memory, emotion, and cognitive effort (see Saby and Marshall, 2012 for review). In the domain of speech perception, Poeppel and his colleagues report in several publications (e.g., Ghitza et al., 2013) that modifications in theta phase measured with magnetoencephalography (MEG) serve as a marker that “parses” syllables.

The goal of the present study was to use MEG, a brain imaging technique that records changes in magnetic fields stemming from neuronal activity, to measure brain oscillatory activity in the theta (4–8 Hz) frequency band during a speech discrimination task in 6- and 12-month-old infants, as well as in adults. Theta brain rhythms are prevalent in EEG during infancy. Adults show theta rhythms, as well as higher frequency brain rhythms such as alpha and beta, which are more prominent in adults than in infants (Saby and Marshall, 2012). Increased theta oscillatory activity has been linked to increases in cognitive demand (Inanaga, 1998) and emotion processing (Hagne, 1972). In infants, increased theta has been observed in response to vowels pronounced using a “motherese,” as opposed to an adult-directed, speech style (Zhang et al., 2011), and with increases in attention (Stroganova et al., 1998; Orekhova et al., 1999; Berger et al., 2006; Bazhenova et al., 2007).

In the current study, we examined developmental changes in theta brain oscillatory activity during the perception of native and non-native speech contrasts, and hypothesized that theta brain rhythms would be sensitive to the transition that occurs in the latter half of the first year. In the sections that follow, we develop an argument that explains our prediction: we argue that the transition in infant speech perception is affected by a change in implicit attention and cognitive effort. Early in infancy, prior to learning which phonetic units are relevant in their particular language, infants implicitly attend to stimuli that are presented with high distributional frequency. This (implicit) strategy assists in the discovery of the specific phonetic units used in their particular language(s). In adulthood, attending to the raw frequency of incoming syllables is not efficient; instead, adults' implicit strategy is driven by their learned native language categories; after learning, non-native categories stand out, and require increased attention and effort, affecting theta rhythms.

To investigate our hypotheses, we utilized a traditional oddball discrimination paradigm to examine theta oscillatory activity using MEG both before (6 months) and after (12 months) the transition in speech perception, as well as in adulthood, in response to: (1) frequent as opposed to infrequent speech syllables, and (2) native as opposed to non-native speech syllables. In infancy, we hypothesized that increased theta relative to baseline activity would be related to the frequency of stimuli, regardless of their language status (native or non-native). In adulthood, we hypothesized that increased theta relative to baseline would be associated with language status (native or non-native), irrespective of the frequency of presentation of the stimuli.

### The transition in developmental speech perception

What do we know about the mechanisms underlying the “sensitive period” for phonetic learning? Research has shown that at least two factors—one computational in nature and the other social in nature—alter phonetic perception during the sensitive period of development in typically developing infants (Kuhl, 2010). Evidence for a computational component stems from studies that show “statistical learning” in infants, a sensitivity to the statistical patterns in language input that have been shown to affect phonetic learning, either through natural language exposure (Kuhl et al., 1992) or through the experimental manipulation of the distributional frequency of phonetic units (Maye et al., 2002, 2008). Studies data show that the potency of distributional frequency in affecting speech perception decreases as early as 10 months of age (Thiessen, 2010; Yoshida et al., 2010). In other words, there is evidence of a waning in the sensitivity to distributional frequency with age, which reduces the effect that a particular stochastic pattern in language has on speech perception, and the waning occurs at the age at which the transition in speech perception occurs. Clearly, in adulthood, exposure to a new pattern of distributional statistics of phonetic units, which occurs whenever adults move to a new country and are exposed to its novel language, does not induce robust phonetic learning, even after extensive training (Flege, 1995).

In addition to a computational component, there is evidence that a social component alters phonetic learning during the sensitive period. Infants exposed for the first time to the statistics of a new language in playful interactive sessions between 9- and 10-months of age showed robust learning of a new Mandarin Chinese phonetic contrast (Kuhl et al., 2003). Social interaction with a live tutor is necessary for this kind of natural language learning to occur: Exposure to the exact same material on the exact same schedule and in the exact same setting from a video resulted in no learning (infants performed equivalently to a control group exposed only to English) (Kuhl et al., 2003). These studies buttress arguments that a sensitive period for phonetic learning exists at about 9 months of age. At 9 months, exposure to a complex natural language via interaction with another human being induces learning (see Kuhl, 2007, for discussion).

Finally, the phonetic learning that occurs during this sensitive period may be critical to language learning. Infants' phonetic learning at this age is strongly linked to their future language growth. Longitudinal studies using behavioral (Tsao et al., 2004; Kuhl et al., 2005) as well as brain measures (event-related potentials, ERPs) (Rivera-Gaxiola et al., 2005; Kuhl et al., 2008), reveal that the ability to discriminate native language phonetic units predicts the growth of language to the age of 30 months, as well as reading readiness at the age of 5 years, independent of socio-economic status of the child's family, and independent of the child's language scores at the age of 30 months (Cardillo Lebedeva and Kuhl, 2009; Cardillo, 2010). These data are correlational in nature; causal relations cannot be assumed. Nevertheless, the data support the idea that phonetic learning during a sensitive period in early development may be an important pathway for initial language learning that aids learning at higher levels of language.

### Mechanisms of developmental change and brain oscillatory activity

Given the potential importance of early phonetic learning, recent research has been focused on identifying the mechanisms that underlie both the timing and the nature of the perceptual narrowing process. Regarding timing, premature infants, who by virtue of early birth have a longer period of exposure to speech by the end of the first year, do not show the transition in speech perception at an earlier age (Pons et al., 2012). This result, plus the data mentioned previously showing new phonetic learning from first-time exposure to a new language at 9 months (Kuhl et al., 2003) suggest that the perceptual narrowing process is not brought about by a prescribed amount of language exposure in the infant's environment, nor by a protracted period of exposure to a language. On the other hand, maternal prenatal exposure to antidepressants (serotonin reuptake inhibitors, SRIs) has been reported to accelerate phonetic development in infants, while untreated maternal depression is reported to slow the timing of the transition in infant speech perception (Weikum et al., 2012). These results, along with previously reviewed findings showing that social interaction promotes phonetic learning in typically developing infants (Kuhl et al., 2003; Conboy and Kuhl, 2011; Kuhl, 2011), and results showing an association between speech perception and the volume of the amygdala—infants with larger right amygdala at 6 months showed lower expressive and receptive language scores at age 2, age 3, and age 4 (Ortiz-Mantilla et al., 2010)—implicates the limbic system and the regulation of motivation and emotion in the transition in phonetic perception (see also Deniz Can et al., 2013).

One candidate mechanism for pan-sensory perceptual narrowing related to social/emotional development is the nascent set of abilities associated with infant attention. Evidence indicating that social interaction is a key skill in language development is of interest because social abilities such as eye gaze following come on line during the putative critical period for phonetic learning in typically developing infants. The ability to attend to another's gaze is a developmental ability that typically appears to emerge between 9 and 12 months of age (Childers and Tomasello, 2002; Gleitman et al., 2005; Hoff, 2006; Meltzoff et al., 2009; Naigles et al., 2009; Csibra, 2010). Posner and Raichle (1994) hypothesized that the onset of an initial nascent form of attentional control during infancy stems from maturation of an anterior attention network. In the domain of speech perception, infants' tendency to show a decline in the perception of non-native phoneme contrasts is significantly correlated with the growth of attentional control skills (Lalonde and Werker, 1995; Hespos and Spelke, 2004; Conboy et al., 2008).

Studies utilizing EEG and MEG suggest that oscillatory brain activity over time reflects changes in attention, learning, and memory. Neural rhythms are hypothesized to indicate synchronized global and local neural networks that operate at different frequencies (Varela et al., 2001; Buzsáki and Draguhn, 2004), and vary with sensory, motor and cognitive (attention and memory) task demands (Klimesch, 1999). Brain rhythms reveal selective activation and inhibition of neural networks involved in sensory and cognitive processing (Knyazev, 2007).

Theoretical work indicates that brain oscillations in the theta band (~4–8 Hz in adults) index the control of attention and cognitive effort in adults involving a wide variety of verbal and non-verbal stimuli. In adults, theta measured with EEG or MEG is linked to focused attention and the encoding of new information (Klimesch, 1999), as well as novelty detection and increased memory load (Jensen and Tesche, 2002; Hsiao et al., 2009). Jensen and Tesche (2002) recorded MEG responses from 10 adult subjects who were asked to retain a list of 1, 3, 5, or 7 visually presented digits during a 3-s retention period. The authors show that during retention, theta activity increased parametrically with the number of items on the list. The results were interpreted as reflecting an increase in the allocation of cognitive resources that are required as the demands on working memory increase. In adults, interpretation of increased theta activity with working memory demands is that the increase reflects enhanced attention (Mizuki et al., 1980; Bruneau et al., 1993; Gevins et al., 1997).

Studies examining theta power are increasing, and theta brain rhythms have been recorded in infants as young as 2 months of age using EEG (see Saby and Marshall, 2012 for review). Existing studies are consistent with adult data and suggest that infant theta also increases when attention increases, either during cognitive (Stroganova et al., 1998; Orekhova et al., 1999; Bell and Wolfe, 2007) or social tasks (Stroganova and Posikera, 1993). Zhang et al. (2011) demonstrated increased theta for simple vowel sounds that reflected an infant-directed (“motherese”) style of speaking as opposed to a more standard adult-directed style.

The topographical distribution of theta in infants is sensitive to the cognitive task or behavior under study (Saby and Marshall, 2012). In a study conducted using EEG, Bell and Wolfe (2007) showed that power in the theta band increases with memory load in 8-month-old infants across the entire scalp. However, when the children were tested again at age 4.5 with age-appropriate working memory tasks, increased theta activity was observed at frontal medial sites only. Previously mentioned studies linked frontal theta to the executive control of attention (Stroganova et al., 1998; Orekhova et al., 1999). Orekhova et al. (1999) measured theta in infants aged 8–11 months while they watched an object, anticipated a partner's appearance in a peek-a-boo game, and during the partner's subsequent appearance in the game. Theta increases were maximal during anticipation of the person's appearance at frontal electrode sites, which, the authors argue, support the idea that theta activity in infants increases during tasks that require sustained attention and particularly the regulation of attention (see also Stroganova et al., 1998).

Orekhova et al. (2006) tested both 10-month-old infants and pre-school children aged 3–6 years old during new toy exploration and social stimulation, and reported increases in theta rhythm and suppression of mu rhythm at both ages during both conditions, arguing that these activities engage attentional networks as reflected by the theta rhythm increase.

Berger et al. (2006) showed increases in theta activity in 7-month-old infants when a violation of expectancy occurred in an arithmetic test involving puppets. The authors interpreted the data as revealing a nascent indicator of executive function, even before infants have real control in self-regulatory processes. In summary, the existing literature suggests that theta oscillations, both in adults and infants, are modulated by tasks that elicit increased attention or cognitive effort.

### Design of the current study

Based on prior data and theory, we hypothesized that the measurement of theta oscillatory rhythms in response to speech syllables that vary in relative *frequency* (frequent vs. infrequent) and *language category* (native vs. non-native) would differ in a particular way across age. Specifically, we hypothesized that the two features of phonetic speech signals (frequency and language) would elicit differential attention and cognitive effort (and therefore, increased theta power) in infants as opposed to adults. In infancy, before phonetic learning has occurred, infant attention (and therefore, theta) would increase for highly frequent phonetic elements, as shown by studies of statistical learning (Maye et al., 2002). In adulthood, after phonetic categories are learned, the status of a phonetic element, that is, whether it is drawn from a native vs. non-native category, would be expected to drive attention and cognitive effort (and therefore, theta).

MEG brain imaging was used to investigate these hypotheses. We measured the brain's theta oscillatory rhythms in response to speech syllables at three ages—6- and 12-months of age, as well as in adulthood. The traditional oddball paradigm allowed us to co-vary the two dimensions of interest (distributional frequency and native vs. non-native) to test our hypotheses. Confirmation of the hypotheses should show that theta brain oscillations vary depending on age. We expected a significant interaction between age and frequency and between age and language category: Infants (but not adults) were predicted to show theta increases for frequent over infrequent stimuli; adults (but not infants) were predicted to show theta increases for non-native relative to native syllables. More specifically, we predicted that early in infancy (6 months) increased relative theta power (RTP) would be observed for frequently presented speech stimuli, regardless of the category (native vs. non-native) from which the sounds were drawn. In contrast, we hypothesized that increased RTP would be observed for non-native speech stimuli in adults, regardless of the frequency with which they are presented. The prediction that non-native stimuli would produce increased theta responses stems from previous work showing that adult's processing of non-native speech stimuli increases the spread and duration of brain activation, effects associated with greater cognitive effort (Zhang et al., 2005, 2009). 12-month-old infants were expected to show a transitional pattern that more closely resembled that of adults than 6-month-old infants.

## Method

### Participants

**Figure 1:**
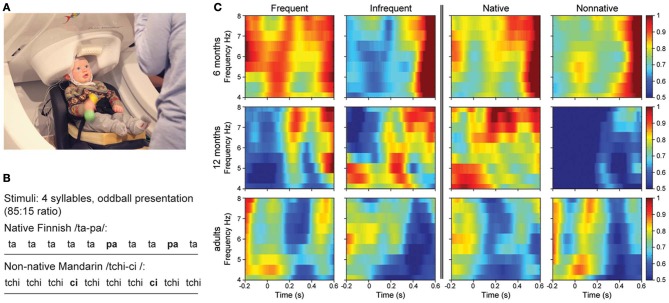
**(A)** Infant in MEG during measurement. **(B)** Stimuli presented in the oddball paradigm, in two conditions, native (upper) and non-native (lower). Bolding reflects infrequently presented stimuli. **(C)** Time-Frequency plots showing the changes in RTP across conditions and groups. Left Panel, Frequency: RTP to frequent and infrequent phonemes collapsed across native and non-native speech sounds as a function of age. Right Panel, Language: RTP to native and non-native phonemes collapsed across freqent and infrequent stimuli as a function of age.

Seventeen healthy full-term Finnish-learning infants were tested at two ages: 6 months of age and 12 months of age in the MEG (see Figure 1A). The 6-month-old infants (*N* = 7) averaged 6.3 months at test (range = 5.15–7.27 months; 3 female); the 12-month-old infants (*N* = 11) averaged 12.27 months at test (range = 9.27–13.2 months; 3 female). An additional 17 infants were excluded due to failure to remain inside the MEG sensor array (2), insufficient data from head position sensors in the MEG (4), failure to complete the two required test conditions (11), or experimenter error (1). Infants were recruited by soliciting families at parent-infant groups and swim lessons in Helsinki, Finland. Written informed consent in accordance with the Research Ethics Board of BioMag Laboratory at Helsinki University Central Hospital and University of Washington was obtained from the parent.

Nine native Finnish-speaking adults (5 female) were tested (mean age = 38.3 years, range = 24.0–57.4 years). Adults were recruited with approved recruitment flyers posted at BioMag Laboratory and in public places in Helsinki, Finland. Adult participants gave written informed consent in accordance with the Research Ethics Board of BioMag Laboratory at Helsinki University Central Hospital and University of Washington.

### Stimuli

Two sets of computer-synthesized syllables were used, one native (Finnish) and one non-native (Mandarin Chinese). The computer synthesized native speech tokens were the Finnish bilabial stop /pa/ and alveolar /ta/ syllables created for use in the current experiment (see Bosseler, 2010 for full description). The computer synthesized male tokens of Mandarin Chinese (alveolo-palatal /tchi/ and fricative /ci/ syllables), were originally created by Kuhl et al. (2003) and used by Kuhl et al. (2005); Tsao et al. (2006), and Kuhl et al. (2008). The stimuli were presented at 65 dBA sound pressure level via a loudspeaker located in front of the participant. Native and non-native sounds were tested in counterbalanced order. Sounds were presented in an oddball paradigm consisting of a standard (0.85 probability), and deviant (0.15 probability) (see Figure 1B). For the native stimuli, /pa/ served as the standard and /ta/ the deviant; for the non-native stimuli, /tchi/ served as the standard and /ci/ the deviant. Sounds were presented with a 1200 ms inter-stimulus-interval (ISI), onset-to-onset. During the experiment, adults were instructed to watch a self-selected silent movie on a screen and ignore the auditory stimuli that were being presented. Infants watched an assistant playing quietly with toys while a silent child-oriented video was displayed behind the assistant.

Borrowed Russian words, which are heard frequently by Finnish people, contain the same alveolo-palatal vs. fricative contrast present in the Mandarin stimuli. Consequently, both the Finnish and Mandarin phonetic contrasts are discriminated by native-Finnish speakers. In pilot studies, we verified that Finnish-speaking adults identified only the Finnish sounds as native to the language using behavioral tests, and that adults could discriminate both the Finnish and Mandarin sounds (Bosseler et al., 2010). As expected, MEG measures of the mismatch negativity (reported in Kuhl et al., in preparation) confirmed that both the Finnish and the Mandarin Chinese contrasts were neurally discriminated by all subject groups: the 6-month-old, 12-month-old, and adult Finnish participants. Discriminatory brain activity to both the native and the non-native contrasts was shown by statistically significant mismatch responses at the source level in auditory cortex (full report in Kuhl et al., in preparation). The goal of the present study was to compare brain rhythms in response to phonetic units varying in frequency and language using phonetic contrasts that are discriminated by all groups of listeners. The ability to discriminate both contrasts allows us to attribute observed theta differences to psychological processes beyond simple discrimination of the stimuli.

### Magnetoencephalography recordings

Auditory evoked magnetic fields (AEF) were recorded with a whole-head array of 306 channels with 102 triple-sensor elements, each with two orthogonal planar gradiometers and one magnetometer (Elekta Oy, Helsinki, Finland) in a magnetically shielded room (Euroshield, Eura, Finland) at the BioMag Laboratory, Helsinki University Central Hospital. The MEG was continuously recorded with a bandpass filter of 0.03–200 Hz and sampled at 600 Hz. Prior to the recordings, four indicator coils were attached to the infant's nylon cap at known locations in an anatomical coordinate system defined by the nasion and the preauricular points. The signals from the coils were used to determine the position of the head inside the helmet. During the experiment, infants were sitting in either a high chair or a car seat. An adult (in addition to the assistant manipulating toys) was in the room sitting to the side of the infant in the MEG during data collection. The duration of each condition was approximately 15 min.

### Data analysis

MEG activity was averaged for each of the 4 stimuli (2 Finnish, 2 Mandarin) offline. Only the pre-deviant standards were used during analyses in order to match the number of deviant and standard epochs. The averaging epochs were taken from 100 ms prior to trigger onset to 1200 ms. Spatiotemporal signal space separation (tSSS) was used to eliminate artifacts arising from sources outside the sensor array such as the heartbeat, limb movements, and other ambient magnetic disturbances (Taulu et al., 2004; Taulu and Simola, 2006). After rejecting artifacts using tSSS, the head position registered for each epoch was used to convert the MEG signals to correspond to a virtual unified (standardized) position within the MEG sensor array for averaging across epochs (Taulu and Kajola, 2005).

### Brain rhythm calculation

The amplitude of single-trial oscillations was calculated using a Fourier transform variant, the Morlet wavelet function of time and frequency, on individual raw data files. A set of wavelets was used with the fundamental frequency ranging from 0 to 30 Hz in steps of 0.5 Hz, using a wavelet width of 7 cycles (Tallon-Baudry et al., 1996). The time-frequency response (TFR) window was symmetrically extended to 1200 ms pre- and post- stimulus onset. The subsequent analysis concentrated on the TFR segment from −100 to 600 ms with respect to stimulus onset to avoid artifact at the ends of the TFR window related to the TFR computation. Changes in power as a function of time were calculated from the single-trial MEG signals. The power change value (ΔP) after the stimulus onset was obtained by computing the change in theta power relative to the 100-ms pre-stimulus baseline. The single-trial data were then averaged separately for the standard native, standard non-native, deviant native, and deviant non-native for each age group. The resulting values are expressed as the change in power relative to the power in a 100-ms pre-stimulus baseline period (Figure 1C). Mean area measurement windows were taken between 0–200 ms, 200–400 ms, and 400–600 ms. Measurement windows were based on previous studies and inspection of individual time-frequency averages and grand averages of each age group.

The SSS method was used to transform the signals of the MEG sensors into magnetostatic multipole moments which were then combined into a total current estimate as given by Equation 18 in Taulu and Kajola (2005). The integral of this estimate was calculated over the whole brain volume to give us a single time-dependent scalar value, reflecting the power of all brain activity. This procedure helps alleviate the reconstruction noise effects related to head position transformations in movement-compensated sensor signals (Medvedovsky et al., 2007). A refinement of the documented SSS algorithm, Foster's optimal inverse (Foster, 1961), was used in the linear transformation of the physical sensor signals to multipole moments to further improve the signal-to-noise ratio.

The amplitude of the multipole moment based total current was then used to calculate activity in the time-frequency domain. In humans, theta recordings during learning and memory tasks have been shown to be widespread across cortical and sub-cortical brain areas (Caplan et al., 2001; Buzsáki, 2005). Although MEG source modeling of theta would have been useful, we were unable to obtain individual infant MRIs in Finland and therefore, completely accurate modeling of the head's conductivity profile was not possible. The aforementioned simple current estimate did not require a priori knowledge of the individual conductivity profile of the infant brain. Thus, the multipole moment method allowed us to avoid the bias associated with source modeling in cases in which the head does not fit a typical conductivity profile as well as estimate RTP across cortical and sub-cortical areas.

## Results

We tested two specific hypotheses: (1) RTP will be greater for Frequent vs. Infrequent stimuli in 6-month-old infants (but not in adults), regardless of language, because infant attention is drawn to high distributional frequency events; (2) RTP will be greater for Non-native vs. Native stimuli in adults (but not in 6-month-old infants), regardless of the frequency of presentation, because non-native stimuli require more attention and cognitive effort. We expected 12-month-old infants to show a transitional pattern that resembled more closely the pattern shown by adults.

Previous work on infants' theta rhythms using fast Fourier transform with narrow frequency bin analysis of EEG data suggests that the frequency range of theta for infants is 3.6–5.6 Hz (Orekhova et al., 1999, 2006). A 4–8 Hz range for theta is widely accepted for adults (see Knyazev, 2007 for review). We calculated RTP using both frequency ranges and similar results were obtained. We report the 4–8 Hz range results here.

Following previous studies (Zhang et al., 2011), we first examined RTP in the 0–200 ms time window in a mixed-model ANOVA with stimulus Frequency (Frequent vs. Infrequent) and Language (Native vs. Non-native) as within subject factors, and Age (6 months, 12 months, and adults) as the between-subjects factor. Greenhouse-Geisser corrections were applied when appropriate and partial eta-squared (η_*p*_^2^) was calculated for main effects and interactions. Planned comparisons were reported as significant at the 0.05 level and Cohen's *d* was calculated for effect sizes.

**Figure 2:**
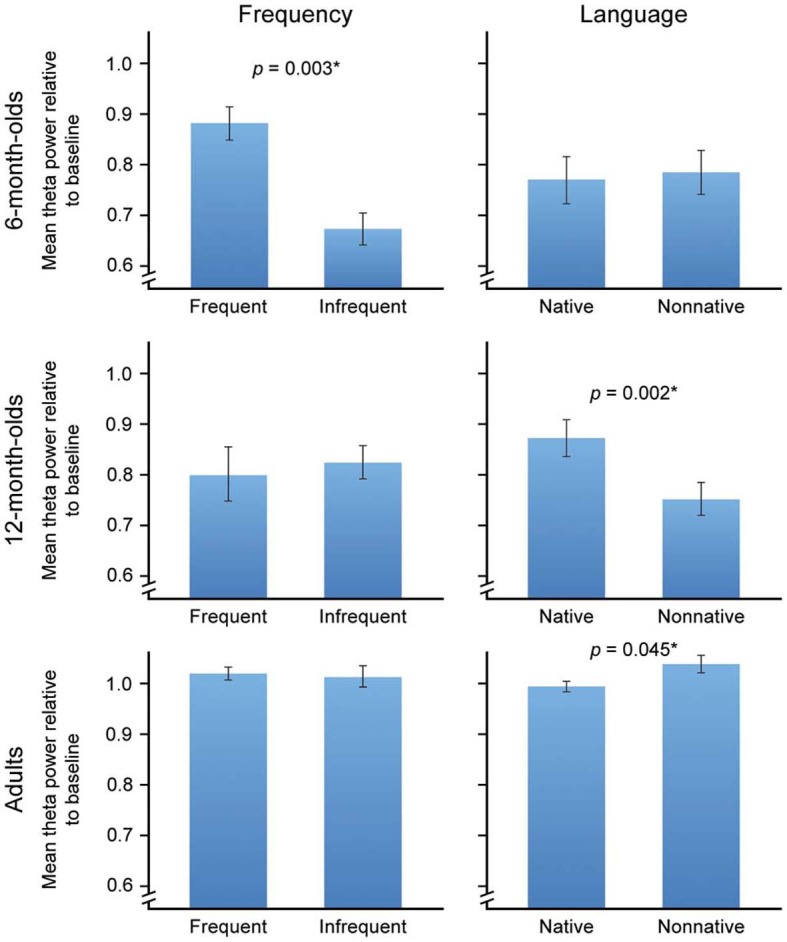
**Left Panel,** Frequency: RTP to frequent and infrequent phonemes collapsed across native and non-native categories as a function of age in the 0–200 ms time window. **Right Panel**, Language: RTP to native and non-native categories collapsed across frequent and infrequent stimuli as a function of age in the 0–200 ms time window. The ^*^ indicates significance level at 0.05 or better. Error bars reflect the standard error.

Theta brain rhythm results were consistent with our predictions. Figure 2 shows the overall RTP at each of the three ages for the factors of Frequency (Figure 2, left column) and Language (Figure 2, right column). A three way repeated-measures ANOVA revealed a significant main effect for the between groups factor of Age, *F*_(2, 24)_ = 25.63, *p* < 0.001, η_*p*_^2^ = 0.68. Tukey HSD revealed that RTP in adults was significantly higher than for the 6- and 12-month-old infants than adults (*p* < 0.001), and did not differ significantly for 6- and 12-month-old infants (*p* = 0.58). A significant main effect was also obtained for Frequency, *F*_(1, 24)_ = 4.34, *p* = 0.048, η_*p*_^2^ = 0.15, indicating higher RTP for Frequent (*M* = 0.9, *SE* = 0.03) vs. Infrequent (*M* = 0.84, *SE* = 0.02) stimuli. There was no main effect for Language, *F*_(1, 24)_ = 0.7002, *p* = 0.41, η_*p*_^2^ = 0.03.

The statistical results of greatest interest to our hypotheses were the predicted significant 2-way interactions. An Age X Frequency interaction, *F*_(2, 24)_ = 5.061, *p* = 0.015, η_*p*_^2^ = 0.30, confirmed our first predicted result: the effect of Frequency was significant only in 6 month olds, *F*_(1, 6)_ = 23.301, *p* = 0.003 η_*p*_^2^ = 0.80 (Figure 2). RTP did not differ significantly to the frequent and infrequent stimuli in 12-month-old infants, *F*_(1, 10)_ = 0.13, *p* = 0.73, or in adults, *F*_(1, 8)_ = 0.06, *p* = 0.81.

As predicted, we also obtained a significant Age X Language interaction, *F*_(2, 24)_ = 4.96, *p* = 0.016, η _*p*_^2^ = 0.29, with the effect of Language significant in adults, *F*_(1, 8)_ = 5.66, *p* = 0.045, η_*p*_^2^ = 0.41 and 12-month-old infants, *F*_(1, 10)_ = 17.294, *p* = 0.002, η _*p*_^2^ = 0.63. In adults, RTP was higher for the Non-native (*M* = 1.04, *SE* = 0.01) vs. Native (*M* = 0.993, *SE* = 0.017), whereas in 12-month-old infants RTP was higher for the Native (*M* = 0.872, *SE* = 0.04) vs. the Non-native (*M* = 0.753, *SE* = 0.03). Six-month-old infants did not show significant differences between the Native (*M* = 0.769, *SE* = 0.47) and Non-native (*M* = 0.783, *SE* = 0.043), *F*_(1, 6)_ = 0.033, *p* = 0.86, stimuli. The three-way interaction was not significant, *p* = 0.47.

### Changes in theta over time

**Figure 3:**
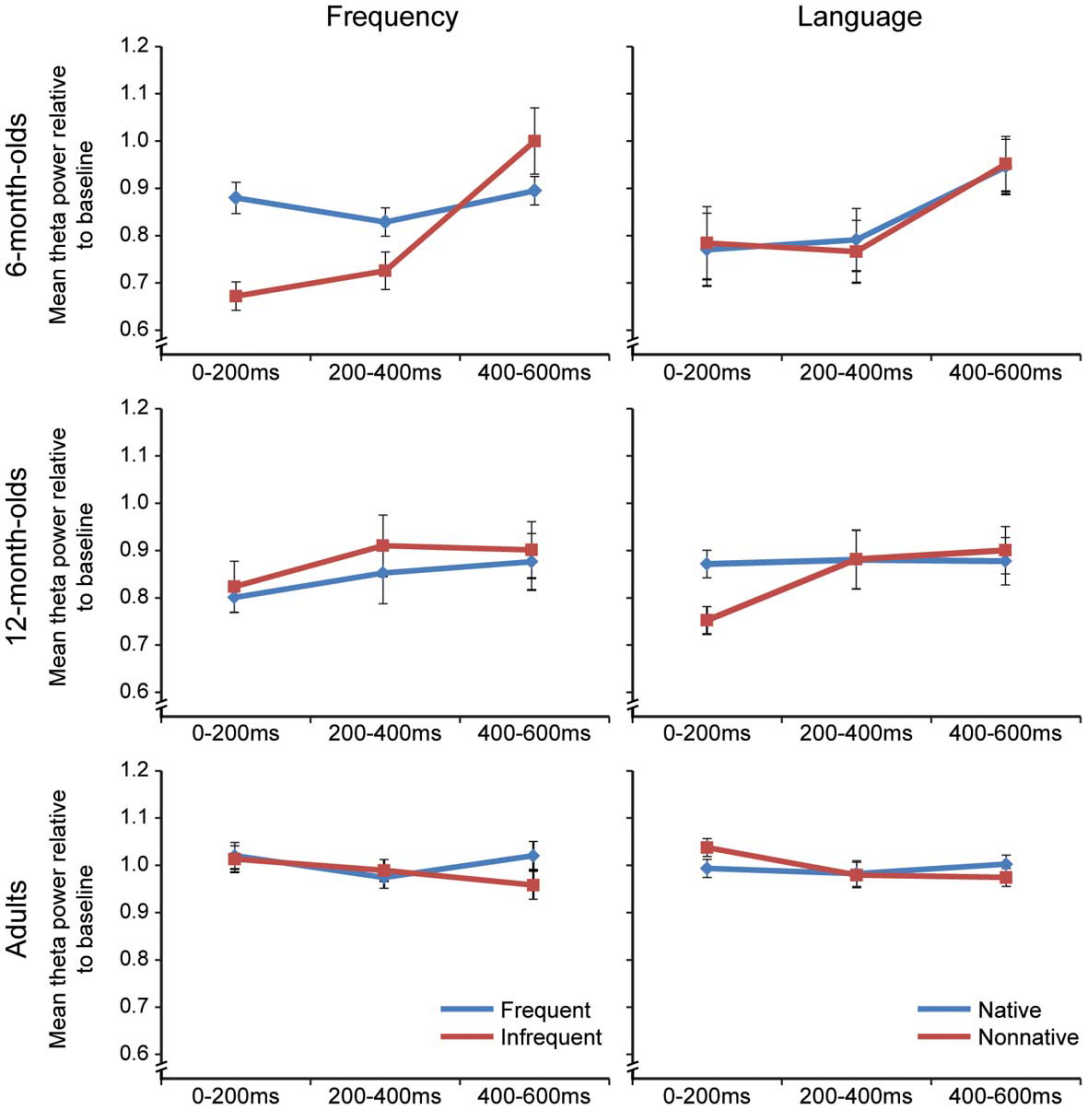
**Left Panel,** Frequency: RTP to frequent and infrequent phonemes collapsed across category dimension (native and non-native categories) in the 0–200, 200–400, and 400–600 ms time windows as a function of age. **Right Panel**, Language: RTP to native and non-native categories collapsed across frequent and infrequent stimuli in the 0–200, 200–400, and 400–600 ms time windows as a function of age. Error bars reflect the standard error.

We had no a priori predictions about the pattern of change in RTP over time at each age, but we were interested in the patterns obtained. The change in RTP over time at each age is shown in Figure 3 for three measurement windows (0–200, 200–400, 400–600 ms). Infants showed the largest changes over time in theta oscillatory activity.

At each age, we conducted a 3-Way ANOVA (Frequency × Language × Window). Looking first at RTP in response to Frequency (Figure 3, left), the results showed that in both 6- and 12- month-old infants, RTP increased from the 1st (0–200) to 3rd (400–600) window: 6 months, *F*_(2, 12)_ = 16.045, *p* = 0.001, η_*p*_^2^ = 0.73; 12 months, *F*_(2, 20)_ = 4.08, *p* = 0.04, η_*p*_^2^ = 0.29. For 6-month-olds, the increase was also significant for the change between the 2nd (200–400) vs. 3rd (400–600) window, *p* = 0.004, *d* = 1.634–0.80. The main effect of Frequency for 6-month-olds indicated higher RTP to the Frequent stimuli, *F*_(1, 6)_ = 5.95, *p* = 0.001, η_*p*_^2^ = 0.50. The Window X Stimulus Frequency interaction, *F*_(2, 12)_ = 7.48, *p* = 0.023, η_*p*_^2^ = 0.56, indicated greater increases over time for Infrequent stimuli, especially between the 2nd and 3rd windows. Follow-up tests showed that the increase to infrequent stimuli was due primarily to Native stimuli (1st to 2nd: *p* = 0.15, *d* = 1.81–0.67; 2nd to 3rd: *p* = 0.01, *d* = 2.65–0.80). The main effect of Frequency and the Window × Frequency interaction were not significant in 12-month-olds or in adults.

For Language (Figure 3, right), RTP increased from the 1st to the 3rd time window in both the 6- and 12- month-old infants, but not in adults, *F*_(2, 16)_ = 2.92, *p* = 0.10, η _*p*_^2^ = 0.27. In 6-month-olds, the increase was significant for both the Native, *p* = 0.006, *d* = 1.50–0.60, and Non-native stimuli, *p* = 0.045, *d* = 1.27, *d* = 0.54. For the 12-month-old infants significant changes in RTP as a function of time occurred for the Non-native stimuli only, indicated by the marginally significant Window × Language interaction, *F*_(2, 20)_ = 3.46, *p* = 0.056, η_*p*_^2^ = 0.26.

## Discussion

The current study focused on oscillatory brain rhythms and the transition in developmental speech perception that all typically developing infants experience between the ages of 6- and 12-months-of-age: A change in phonetic perception that alters the initial language-general mode of perception to one that is language specific. This transition in infant perception has been widely reported across cultures and occurs during a putative “critical” or “sensitive” period in the development of language (Kuhl et al., 2005; Kuhl, 2010; Peña et al., 2012; Weikum et al., 2012). In the current study, our approach was to employ a spectral analysis of MEG-derived neuromagnetic signals. The brain's oscillatory rhythms have been associated in previous studies with changes in stimuli that evoke increased attention and/or cognitive effort.

We focused on the theta brain rhythm (4–8 Hz) because it has often been recorded in infants, as well as adults, and because we hypothesized that changes in implicit attention and cognitive effort, would be associated with the transition in phonetic perception. To our knowledge this is the first time that MEG brain imaging has been used in a study of oscillatory brain activity in response to speech in infants; it is also the first study of oscillatory activity using EEG or MEG in which developmental change from infancy to adulthood is examined.

We hypothesized that theta oscillatory rhythms would increase differentially as a function of age, and more importantly, that theta would vary as a function of two different aspects of the speech stimuli manipulated in the current experiment: (1) distributional frequency, and (2) status as native- as opposed to non-native phonetic stimuli. Interactions between age and frequency and between age and language were predicted: 6-month-old infants (but not adults) were expected to show theta increases for frequent over infrequent stimuli, regardless of language; adults (but not 6-month-old infants) were expected to show significant theta increases for non-native relative to native syllables, regardless of frequency of presentation. 12-month-old infants were expected to show a transitional pattern that more closely resembled that of adults than 6-month-old infants.

Our results confirmed these hypotheses: Theta power in 6-month-old infants was higher in response to frequently presented stimuli, with no significant differences for native as opposed to non-native syllables. Attending to distributional frequency in an array of speech stimuli is very efficient in promoting phonetic learning during infancy, because this probability statistic reveals information that assists the identification of the phonetic units that signal meaningful differences between words. Studies show that language input contains robust distributional frequency information about the phonetic units that distinguish words in the language (for evidence from Japanese, see Werker et al., 2007). Increases in theta oscillatory brain activity to frequently presented syllables may thus, be a biomarker of infant attention to this feature.

In adulthood, theta increases are *not* driven by the frequency with which syllables are presented, but instead by the category (native or non-native) of the syllables. In adults, we observed theta increases in response to non-native as opposed to native syllables, and we attribute this result to the fact that processing non-native phonetic syllables demands greater attention and cognitive effort (see Zhang et al., 2009). Adults' perception of speech is governed by learned categories, and processing is highly automatic. Attention is automatically drawn to syllables that do not belong to learned categories; they require more cognitive effort to process.

The pattern in 12-month-old infants more closely resembled that of adults in that theta change is not driven by the frequency of presentation, and instead by language. However, and interestingly, 12-month-old infants showed higher theta rhythms to native syllables, whereas adults showed increased theta to non-native syllables. At 12 months of age, infants, for the first time, are attending to the detailed characteristics of native language phonetic units, and developing representations in memory of these phonetic units. We interpret this finding as evidence that during the initial transition to native-language processing, when native language learning has begun but is still incomplete, infants' nascent attentional network is directed to the acoustic events that signal meaningful word differences, requiring increased attention and cognitive effort. In adulthood, when phonetic processing has become more automatic, native syllables are processed without effort, and non-native syllables require more attention and effort.

Thus, our data suggest that development, as revealed by theta power, may involve two transitions in perception: the first involves a shift in attention from the acoustic speech signals that occur most frequently, to the acoustic events that have native-language status. Once native-language processing becomes more automatic, as it is in adults, then attention is drawn toward syllables that do not belong to a known category, in this case, a non-native syllable. The fact that adults do not attend to highly frequent events, as they once did in infancy, restricts their abilities to learn new phonetic material. Moving to a new country as an adult, and listening for months or even years to the distributional statistics of a new language, does not lead to robust learning of the phonetics of a new language (Flege, 1995).

The use of MEG in the current study allowed us to observe the temporal unfolding of theta oscillatory activity for the first time. Infants showed more dramatic changes over the 600 ms temporal window when compared to adults, who were remarkably stable in the responses over the 600 ms period. Comparing the two infant groups, 6-month-olds showed more change over time than 12-month-olds, with greater increases to infrequent stimuli, suggesting perhaps that more time is needed to attend to infrequent stimuli. We assume that changes in theta over the 600 ms temporal window we investigated could reflect the value for infants of a longer period during which the stimulus can be analyzed; therefore, increases in theta over time are observed rather than decreases over time. In the 12-month-olds, increases occurred to non-native stimuli over time, perhaps reflecting that additional time increases the attention to non-native stimuli for infants who are acquiring native-language categories.

It is of interest for future studies that work by Poeppel and his colleagues using MEG measures (e.g., Ghitza et al., 2013) indicates that, in adults, theta phase information “parses” syllables in a speech stream, which would aid infant speech processing. Future developmental studies can be directed to investigate theta phase, as well as to investigate other frequency bands, using both power as well as phase measurements, to more fully understand how brain oscillatory rhythms change with age and experience to language stimuli.

### An implicit learning process involving attention

Collectively, the data from the present experiment suggest that theta oscillatory brain activity indexes an implicit learning process that entails a shift between infancy and adulthood in the patterns of speech stimuli that induce attention. The patterns we observed in theta oscillatory activity with age mirror the timing observed in behavioral studies on the transition in developmental speech perception. Theta oscillatory activity has been linked in previous studies outside the domain of speech to changes in attention and cognitive effort, and previous interpretations of theta brain rhythms are consistent with our findings. Infant theta increased for frequently presented stimuli, and this is consistent with literature showing that infants are statistical learners in the early period (see Maye et al., 2002). Infants are drawn to phonetic stimuli in their environment that occur frequently, and this has been shown experimentally to assist phonetic learning. In adults, attention to frequently occurring syllables could reduce the stability of the phonetic categories learned in infancy, which are expected to allow efficient speech processing all one's life. For adults, attention is driven by category knowledge—the recognition that a stimulus is a native vs. a non-native syllable. The results of the current study confirm that adults' theta increases are driven by the syllable category (native vs. non-native), rather than its frequency, with non-native syllables requiring more attention and cognitive effort. As expected, 12-month-old infants are in transition. Theta in 12-month-olds is no longer driven by frequency, and is instead driven by language; however, unlike adults, syllables representing the native category led to increased theta. Infants at 12 months are in the process of locking in learned phonetic categories, resulting in greater attention to native syllables. During the second 200 ms time window (200–400 ms), 12-month-olds begin to resemble adults, showing greater theta increases to the non-native syllables.

Thus, far in the discussion we have interpreted our results in terms of theta oscillatory activity indicating changes in attention brought about by development and experience. But the kinds of changes in attention that we propose are not under the control of the participant. The changes in attention we describe are part of an implicit learning process, one that is not under conscious control. Infants' attentional networks are not fully developed (see discussion by Diamond, 1990, 1994; Berger et al., 2006). Even in adults, theta oscillations do not reflect conscious attentional strategies. The learning process for language guides attentional shifts toward high probability events early in development because probabilistic information reveals structure in language input. In adulthood, attention to frequent stimuli would render learned categories unstable. Brain oscillatory activity indexes these higher-level psychological processes.

Infants' sensitivity to probabilistic events is highly conducive to language acquisition because, prior to learning, the elementary units (phonemes and words) that are critical to language are unknown to infants—attention to the probabilistic information in language input identifies the critical elements (phonemes and words) thus, supporting learning. In adulthood, the brain has developed neural networks that specialize in the analysis of native language patterns of phonemes and words, making analysis more automatic. Attentional demands thus, increase for non-native, rather than native, speech signals (see also Zhang et al., 2005, 2009). Continuing research on theta brain rhythms in typically developing infants and young children, as well as those with developmental difficulties, will advance our understanding of the relationship between attention, executive control, and language acquisition. The measurement of the brain's oscillatory rhythms goes beyond what behavioral studies can reveal.

### Limitations of the present study

In the present study, theta brain rhythms were measured using the overall amplitude of total current based on multipole moments, which provided a whole-brain assessment (a “virtual” sensor) that we submitted to spectral analysis. In future studies, improved noise-reduction algorithms that are currently being developed will improve our ability to track the temporal dynamics of brain activation in both infants and adults. In addition, ongoing work in optimizing the movement compensation algorithm with respect to reconstruction noise will ensure a robust sensor-level analysis even with subjects whose movements are larger than the current limits considered acceptable for reliable analysis (Medvedovsky et al., 2007). Furthermore, the use of magnetic resonance imaging (MRI) of infant participants, or the use of age-specific infant average head models, which we are developing (see Akiyama et al., 2013), would allow brain oscillatory activity to be localized in the infant brain with improved accuracy. Localization of theta brain activity would provide information about hemispheric differences in the generators of this activity across age, as well as the location of brain activation that accompanies the observed shift in theta related to stimulus characteristics—from frequent stimuli in infancy to non-native stimuli in adulthood.

## Conclusions

In the present study, changes in theta brain rhythms were shown to index the universally observed developmental change in speech that narrows infants' perception, a transition in development that has been posited as necessary for language acquisition (Kuhl, 2010). We demonstrate that infant theta oscillations are driven by the distributional frequency of speech events, whereas adult theta oscillations are driven by category knowledge. We posit that these changes in brain oscillations reflect an implicit learning process in which a shift in attention occurs with development and experience: early in infancy, before phonetic learning has occurred, attention is driven by event frequency; once phonetic learning occurs, attention is driven by category knowledge. This learning algorithm, we posit, is based on the brain's implicit learning systems and attentional mechanisms, and the learning algorithm causes the transition in speech perception that occurs before the end of the first year. Whether the timing of the transition in speech perception is governed by maturation, experience, the emergence of other cognitive skills (e.g., social cognition), or a combination of these factors, remains to be determined. Regardless of these future questions, however, the present study demonstrates the utility of theta brain oscillatory activity as an index of a shift in attention from frequent events to learned category knowledge that will be a helpful tool in future studies.

Future studies could examine theta oscillatory rhythms using other stimuli, such as faces, music, and multi-modal speech stimuli (Pascalis et al., 2002, 2005; Hannon et al., 2005; Lewkowicz and Ghazanfar, 2006). All of these stimuli have been reported to show perceptual narrowing in the developmental timeframe during which the transition in infant speech perception occurs. Theta rhythm analyses will reveal either parallels or distinctions across stimulus domains, supporting either a pan-sensory domain-general learning process as the instigator of perceptual narrowing, or a learning process that is highly specific and dedicated to language learning.

### Conflict of interest statement

The authors declare that the research was conducted in the absence of any commercial or financial relationships that could be construed as a potential conflict of interest.
